# The associations between biological markers of aging and appetite loss across adulthood: retrospective case–control data from the INSPIRE-T study

**DOI:** 10.1007/s11357-025-01691-w

**Published:** 2025-05-10

**Authors:** Annelie Turesson, Afsaneh Koochek, Margaretha Nydahl, Jean-Marc Lemaitre, Paul Bensadoun, Laurent O. Martinez, Sophie Guyonnet, Yves Rolland, Bruno Vellas, Philipe De Souto Barreto, The IHU HealthAge INSPIRE/Open Science group, Lauréane Brigitte, Agathe Milhet, Elodie Paez, Emeline Muller, Sabine Le Floch, Catherine Takeda, Catherine Faisant, Françoise Lala, Gabor Abellan Van Kan, Zara Steinmeyer, Antoine Piau, Tony Macaron, Davide Angioni, Pierre-Jean Ousset, Mélanie Comté, Nathalie Daniaud, Fanny Boissou-Parachaud, Sandrine Andrieu, Christelle Cantet, Fabien Pillard, Marie Faruch, Pierre Payoux, Catherine Takeda, Neda Tavassoli, Marie Dorard, Bénédicte Razat, Camille Champigny, Cédric Dray, Jean-Philippe Pradère, Angelo Parini, Yohan Santin, Dominique Langin, Pierre Gourdy, Anne Bouloumié, Nicolas Fazilleau, Roland Liblau, Jean-Charles Guéry, Michel Simon, Nicolas Gaudenzio, Luciana Bostan, Hicham El Costa, Nabila Jabrane Ferrat, Philippe Valet, Isabelle Ader, Valérie Planat, Louis Casteilla, Patrice Peran, Cyrille Delpierre, Claire Rampon, Noelie Davezac, Bruno Guiard, Nathalie Vergnolle, Jean-Paul Motta, Sara Djebali, Pauline Floch, Céline Deraison, Chrystelle Bonnart, Jean-Emmanuel Sarry, Nicola Coley, Jessica Pontary

**Affiliations:** 1 Department of Food Studies, Nutrition and Dietetics, Uppsala University, Uppsala, Sweden; 2 IHU HealthAge, Toulouse, France; 3 INSERM IRMB UMR1183, Hôpital Saint Eloi, University of Montpellier, Montpellier, France; 4 LiMitAging, Institute of Metabolic and Cardiovascular Diseases (I2MC), University of Toulouse, INSERM, University of Toulouse - Paul Sabatier (UPS), UMR1297 Toulouse, France; 5 CERPOP UMR 1295, University of Toulouse, INSERM, UPS, Toulouse, France; 6 Institute On Aging, Toulouse University Hospital (CHU Toulouse), Toulouse, France

**Keywords:** Biological aging, Longevity, Aging clocks, Anorexia of aging

## Abstract

Appetite loss is a common clinical condition in older adulthood, but how this condition associates with biological aging remains unknown. The present study aims to examine the associations of biological aging markers with appetite loss in community-dwelling people aged 21 to 102 years. This retrospective case–control study used baseline data from the INSPIRE-T cohort in Toulouse, France. Each of the 49 cases with appetite loss was sex- and age-matched to two controls without appetite loss (*n* = 147; median age of 79 years, interquartile range: 19.5; 67% women). Appetite loss was assessed using a single yes–no question from the World Health Organization´s Integrated Care for Older People screening tool. Biomarkers (first- and second-generation DNA methylation-based epigenetic clocks [Horvath, Hannum, PhenoAge, and GrimAge], the inflammatory aging clock iAge, and Adenosine triphosphatase inhibitory factor 1—IF1) were derived from blood samples. Logistic regression analyzed the associations of these markers with appetite loss. In fully adjusted models, accelerated aging using GrimAge was the only biomarker associated with appetite loss (Odds Ratio = 1.21, 95% Confidence Interval: 1.03, 1.43). When stratified by age (≤ 65 years vs. > 65 years) and sex, this association remained significant only in individuals over 65 years and men. Future research is needed to explore the potential mechanisms involved, as well as how other biological drivers of aging (e.g., cell senescence, deregulated nutrient sensing) relate to appetite loss.

**Supplementary Information:**

The online version contains supplementary material available at 10.1007/s11357-025-01691-w.

## Introduction

Loss of appetite during aging, also referred to as anorexia of aging, is associated with adverse health outcomes, including depression, undernutrition, reduced muscle function, frailty, and increased mortality risk [1, 2]. Its high prevalence, around 15–30% in different populations of older adults, and significant health implications underscore the vital role that poor appetite plays in the well-being of older people [1–3]. Although it may be considered an age-related clinical condition, the extent to which biological aging processes determine appetite loss is still unknown. Understanding the biological aging-appetite loss interplay may open the avenue for new treatment strategies since, to date, there is no drug approved to treat anorexia of aging.

Aging is a complex process characterized by molecular and cellular damage over time, leading to functional decline and chronic diseases [4, 5]. Epigenetic changes are considered one of the drivers of aging, and several DNA methylations clocks have been proposed to assess the pace of aging, such as Horvath´s [6], Hannum´s [7], PhenoAge [8] and GrimAge [9] clocks (the two formers are 1 st generation; the two latter, 2nd generation clocks). More recently, an inflammatory clock (iAge) has been proposed [10] as a marker of the pace of aging. Indeed, chronic inflammation is a major determinant of aging, and inflammatory markers are already known to impact appetite regulation, as inflammation can affect signalling pathways involved in hunger [11]. Mitochondrial function is a major determinant of aging. As a potential marker of mitochondrial bioenergetics, the Adenosine triphosphatase (ATPase) inhibitory factor 1 (IF1), a natural inhibitor of mitochondrial ATP synthase that regulates oxidative phosphorylation activity [12] may play a role in the pace of aging [13, 14]. Although a few previous studies have examined the associations of inflammation [11, 15] and IF1 [16] with appetite loss during aging, no studies have explored the associations of biological aging clocks and appetite loss. Furthermore, how the appetite-biological aging association behaves across adulthood (from young to very old adults) and by sex is completely unknown.

The primary objective of this study was to examine the associations of biological aging markers, including epigenetic clocks, inflammatory clock, and IF1, with appetite loss in community-dwelling people aged 21 to 102 years. Secondary objectives were to explore these associations stratified by age and sex. We hypothesized that greater accelerated biological aging would be associated with appetite loss.

## Method

The INSPIRE-T cohort is conducted in accordance with the Declaration of Helsinki, and the study protocol received approval from the French Ethical Committee (CPP Ouest V) in Rennes in October 2019. All the participants signed an informed consent. The INSPIRE-T study is registered in a publicly accessible database (https://clinicaltrials.gov/) under the registration number NCT04224038.

### Study population

**Fig. 1:**
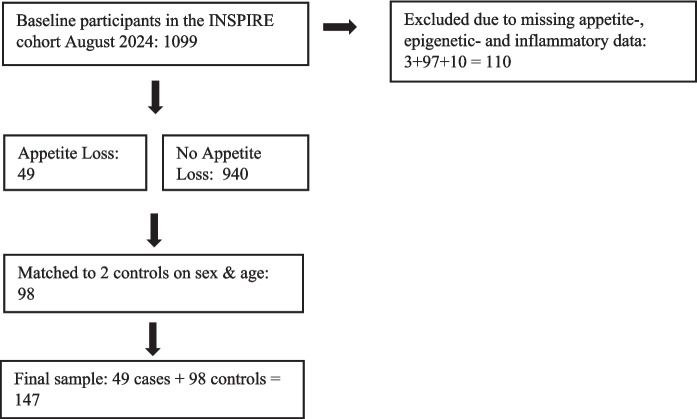
Flowchart of participant inclusion and selection

This retrospective case–control study utilized baseline data from the INSPIRE-T cohort in Toulouse, France. The INSPIRE-T cohort is an ongoing observational study with a planned 10-year follow-up, specifically designed to investigate biomarkers of aging and intrinsic capacity in humans. Detailed descriptions of the cohort are available elsewhere [17, 18]. By 2024, 49 individuals in the INPISIRE-T cohort have declared to suffer from appetite loss and had available data for biomarkers of aging, in particular epigenetic and inflammatory clocks as well as IF1. The 49 cases were matched in a 1:2 ratio to controls, resulting in 98 controls. The final sample included 147 participants with a median age of 79 years (IQR = 19.5) and 67% women (see Fig. 1).

### Case–control matching

The variables used for matching were sex and age. The 49 cases were perfectly matched for sex, with 45 perfectly matched for age. Three cases were matched with controls differing by one year in age, while one case, a 102-year-old male, was matched with two controls aged 90 (closest matching-control available). Detailed information about the matching variables is presented in Table A1 in the supplemental material.

### Outcome measure

The outcome of this study was appetite loss, assessed using the following yes–no question from the World Health Organization´s Integrated Care for Older People (ICOPE) screening tool [19]: “Have you experienced loss of appetite?”.

### Exposure

The exposures were epigenetic and inflammatory aging clocks, along with IF1.

The epigenetic clocks were derived from extracted DNA from blood samples in the INSPIRE-T cohort using the Qiagen DNeasy Blood & Tissue kit (Qiagen N.V., Vienlo, Netherlands). The Genomic DNA was then subjected to bisulfate conversion and DNA methylation was analyzed using the EPIC Infinium array (Illumina Inc., San Diego, USA), following the manufacturer´s guidelines. Methylation levels for each CpG site, ranging from 0 (fully unmethylated) to 1 (fully methylated) were calculated using Partek® Genomics Suite® software (Partek Inc., Chesterfield, USA). The epigenetic clocks Horvath [6], Hannum [7], and PhenoAge [8] were calculated using Methyl clock R package [20] and the GrimAge clock was calculated using *minifi* R package as described in the original paper [9].

For the inflammatory clock [10], blood samples from the INSPIRE-T cohort were diluted threefold in Luminex assay buffer and analyzed using a Luminex L200 with a custom ProCartaPlex Luminex kit (Thermo Fisher, Santa Clara, USA). Quality control was maintained by adding Assay Chex Beads (Radix BioSolutions, Georgetown, USA) to each well. Samples were processed in duplicate and incubated overnight with the Luminex beads. The adjusted mean intensity values were calculated and averaged for each sample. To align analyte levels with the Stanford 1 KIP data, protein standards from the Luminex kit were used on each plate and the same standards were run on a bridge plate with previously analyzed 1 KIP samples. All analyses were based on the average adjusted mean intensity values. A linear regression model was created using the top five analytes (CCL11, CXCL1, CXCL9, IFNG, and TRAIL) that best predicted iAge in the INSPIRE-T cohort. Both epigenetic and iAge acceleration were derived as the residuals obtained by regressing chronological age on epigenetic age, adjusted for cell number.

Additionally, IF1 levels in the blood were measured directly using a biological assay. Samples were prepared using the ProteinWorks™ eXpress kit by heating them in a digestion buffer containing labeled peptides and RapidGest detergent. They were then reduced with dithiothreitol, alkylated with iodoacetamide, and digested with trypsin. Digestion was stopped and followed by centrifugation to remove precipitates. The supernatant was cleaned and the fractions were dried under nitrogen before being reconstituted and injected into the LC–MS/MS system. Analysis was performed using a Xevo® TQD mass spectrometer with an electrospray ionization interface and an Acquity H-Class® UPLC device (Waters Corporation). Data were acquired and processed using MassLynx® and TargetLynx® software. A more detailed explanation can be found elsewhere [21]. Plasma levels were assessed in a subsample of 121 participants due to missing data for the remaining participants.

### Confounders

The included covariates were: Body Mass Index (BMI) (kg/m^2^, continuous), self-reported number of medications per day (continuous), depressive symptoms (categorical), recent weight loss (categorical), and cognitive impairment (categorical). The latter three were assessed using the World Health Organization´s ICOPE screening tool [19]. Depressive symptoms were evaluated with two questions: “Over the past two weeks, have you been bothered by feeling down, depressed or hopeless?” and “Over the past two weeks, have you been bothered by having little interest or pleasure in doing things?” A “yes” response to either question was sufficient to indicate depressive symptoms. Recent weight loss was assessed by the question: “Have you unintentionally lost more than 3 kg over the last three months?” (yes/no). Cognitive impairment was assessed using a memory test (recalling three words) and an orientation test (stating the full date and location of the assessment). Any error was considered as having cognitive impairment. It should be mentioned that covariates from the ICOPE screening tool were preferred instead of full-length scales for cognitive function and depressive symptoms to avoid losing cases with missing data in full-length scales; this was an important choice given the small number of cases.

### Statistical analyses

Descriptive statistics (median and interquartile range (IQR) and frequency (%), as appropriate) were used to characterize the groups. Between-group comparisons were performed using the Chi-Square Test or Fisher’s Exact Test, and the Wilcoxon Rank-Sum Test, as appropriate.

Odds ratios and 95% confidence intervals were estimated using a multivariate conditional logistic regression model to assess the association between appetite loss (dependent variable) and aging biomarkers (independent variables). Both unadjusted and adjusted models were performed to evaluate the associations of each biomarker (separate models for each biomarker). The adjusted models included the confounders described above: BMI, medications, depressive signs, weight loss, and cognitive impairment. Since sex was perfectly matched, and age was almost perfectly matched, these were excluded as covariates in the conditional logistic regression model. This method automatically adjusts for matched variables by conditioning on the matched sets, ensuring that any confounding related to these factors is addressed through matching [22]. For the secondary objectives, associations were analysed separately by sex and age using logistic regression. Models were adjusted for age or sex, depending on the stratification, as well as for BMI, medications, weight loss, depressive symptoms, and cognitive impairment. All statistical analyses were conducted using the statistical software Stata version 18.0 (Stata Corp, College Station, Texas 77,845 USA).

## Results

The characteristics of the included 147 participants per group are presented in Table 1. People with appetite loss reported taking more medications (6 vs. 2 per day), more often had recent weight loss (20% vs. 6%) and depressive symptoms (67% vs. 26%) than those without appetite loss. Regarding the biomarkers of aging, differences between the groups were observed in age acceleration for PhenoAge and GrimAge, where cases showed accelerated aging while controls showed decelerated aging for both clocks.

**Table 1:** Differences in Characteristics, Epigenetic and Inflammatory Clocks and ATPase Inhibitory Factor 1 Between Cases and Controls (*n* = 147)

Variable	Cases (*n* = 49)	Controls (*n* = 98)	*P*-value
Age *	79 (19)	79 (20)	0.980
Sex *			1.000
Women	33 (67.35%)	66 (67.35%)	
Body Mass Index	24.8 (6.29)	24.5 (5.24)	0.993
Medications/Day	6 (7)	2 (5)	< 0.001
Recent weight loss			0.009
> 3 kg loss (last 3 months)	10 (20.41%)	6 (6.12%)	
No weight loss	39 (79.59%)	92 (93.88%)	
Depressive signs			< 0.001
Yes	33 (67.35%)	25 (25.51%)	
No	16 (32.65%)	73 (74.49%)	
Cognitive impairment			0.571
Yes	2 (4.08%)	5 (5.10%)	
No	47 (95.92%)	93 (94.90%)	
Biomarker of aging			
Horvath Age Acceleration^1^	−0.49 (6.57)	−0.44 (5.82)	0.992
Hannum Age Acceleration^2^	−0.85 (5.57)	−0.77 (4.75)	0.495
PhenoAge acceleration^3^	0.91 (6.32)	−0.79 (6.80)	0.043
GrimAge Age Acceleration^4^	1.33 (7.25)	−0.89 (3.70)^a^	0.016
Inflammatory Age acceleration^5^	2.84 (14.27)	1.18 (13.65)	0.386
ATPase Inhibitory Factor 1^6^ (ng/mL)	490.6 (322.9) ^c^	507.3 (257.9) ^b^	0.954

^a^*n* = 97 people in controls, ^b^ *n* = 82 people in controls, ^c^ *n* = 39 people in cases

Chi-Square Test and Fisher´s Exact Test (cell frequency < 5) were used for categorical variables (n (%)). Wilcoxon Rank-Sum Test (median (interquartile range) was used for continuous variables (age, BMI, medications, and biomarkers). * Cases and controls matched on age and sex. ^1^ Horvath Age acceleration, residuals after regressing chronological age on epigenetic age (adjusted for cell number), ^2^ Hannum Age acceleration, residuals after regressing chronological age on epigenetic age (adjusted for cell number), ^3^ PhenoAge acceleration, residuals after regressing chronological age on epigenetic age (adjusted for cell number), ^4^ GrimAge acceleration, residuals after regressing chronological age on epigenetic age, ^5^ Inflammatory Age acceleration, residuals after regressing chronological age on inflammatory age, ^6^ ATPase Inhibitory Factor 1 (IF1), measured in ng/mL in a subsample of 121 participants

### Associations between biomarkers of aging and appetite loss

Table 2 shows the results for unadjusted and adjusted conditional logistic regressions. In the unadjusted analysis, significant associations were found between appetite loss and age acceleration for both PhenoAge and GrimAge. The association between appetite loss and accelerated aging remained significant for GrimAge after adjustments (Odds Ratio = 1.21, 95% Confidence Interval: 1.03, 1.43).

**Table 2:** Associations between epigenetic and inflammatory clocks, ATPase inhibitory factor 1, and appetite loss

	Unadjusted Model N cases = 49, N controls = 98			Adjusted Model N cases = 49, N controls = 98		
Biomarker of aging	Odds Ratio	95% CI for Odds Ratio	*P*-value	Odds Ratio	95% CI for Odds Ratio	*P*-value
Horvath Age Acceleration^1^	0.994	0.926, 1.067	0.868	1.005	0.915, 1.104	0.919
Hannum Age Acceleration^2^	0.956	0.874, 1.046	0.326	0.988	0.870, 1.123	0.856
PhenoAge Acceleration^3^	1.074	1.000, 1.154	0.049	1.062	0.969, 1.163	0.198
GrimAge Acceleration^4^	1.191^a^	1.056, 1.343	0.004	1.212^a^	1.028, 1.428	0.022
Inflammatory Age Acceleration^5^	1.020	0.979, 1.063	0.339	1.050	0.991, 1.112	0.098
ATPase Inhibitory Factor 1^6^	1.000	0.999, 1.002	0.280	1.001	0.999, 1.003	0.244

CI = Confidence Interval. ^a^ n cases = 49, n controls = 97. ^1^ Horvath Age acceleration, residuals after regressing chronological age on epigenetic age (adjusted for cell number), ^2^ Hannum Age acceleration, residuals after regressing chronological age on epigenetic age (adjusted for cell number), ^3^ PhenoAge acceleration, residuals after regressing chronological age on epigenetic age (adjusted for cell number), ^4^ GrimAge acceleration, residuals after regressing chronological age on epigenetic age, ^5^ Inflammatory Age acceleration, residuals after regressing chronological age on inflammatory age, ^6^ ATPase Inhibitory Factor 1 (IF1), measured in ng/mL in a subsample of 105 participants

(Conditional Logistic Regression, Adjusted for Body Mass Index, Medications, Weight Loss, Depression Severity and Cognitive Impairment)

### Age-stratified associations between biomarkers of aging and appetite loss

Table 3 presents the results for unadjusted and adjusted logistic regression, stratified by age (≤ 65 years and > 65 years). In the unadjusted model, an association between appetite loss and accelerated aging for GrimAge was found only in people over 65 years old, and this remained significant after adjustments (Odds Ratio = 1.32, 95% Confidence Interval: 1.09, 1.60). No significant associations were found in people ≤ 65 years.

**Table 3:** Age-stratified associations between epigenetic and inflammatory clocks, atpase inhibitory factor 1, and appetite loss

Biomarker	Model	Age group	Odds Ratio	95% Confidence Interval	*P*-value
Horvath Age Acceleration^1^	Model 1	≤ 65 years	1.066	0.918, 1.238	0.400
		> 65 years	0.975	0.900, 1.055	0.526
	Model 2	≤ 65 years	1.041	0.871, 1.245	0.659
		> 65 years	0.978	0.893, 1.070	0.623
	Model 3	≤ 65 years	1.029	0.847, 1.250	0.775
		> 65 years	0.990	0.893, 1.097	0.848
Hannum Age Acceleration^2^	Model 1	≤ 65 years	1.158	0.913, 1.468	0.227
		> 65 years	0.921	0.830, 1.021	0.116
	Model 2	≤ 65 years	1.171	0.884, 1.550	0.272
		> 65 years	0.926	0.825, 1.039	0.191
	Model 3	≤ 65 years	1.168	0.873, 1.563	0.295
		> 65 years	0.964	0.838, 1.108	0.603
PhenoAge Acceleration^3^	Model 1	≤ 65 years	1.110	0.936, 1.317	0.231
		> 65 years	1.061	0.984, 1.143	0.121
	Model 2	≤ 65 years	1.060	0.875, 1.284	0.551
		> 65 years	1.071	0.987, 1.162	0.102
	Model 3	≤ 65 years	1.090	0.883, 1.347	0.421
		> 65 years	1.067	0.973, 1.169	0.169
GrimAge Acceleration^4^	Model 1	≤ 65 years	1.041	0.854, 1.270	0.691
		> 65 years ^a^	1.207	1.062, 1.371	0.004
	Model 2	≤ 65 years	0.954	0.754, 1.207	0.693
		> 65 years ^a^	1.194	1.034, 1.379	0.016
	Model 3	≤ 65 years	0.995	0.763, 1.299	0.973
		> 65 years ^a^	1.319	1.088, 1.599	0.005
Inflammatory Age Acceleration^5^	Model 1	≤ 65 years	0.991	0.906, 1.085	0.852
		> 65 years	1.027	0.981, 1.074	0.255
	Model 2	≤ 65 years	1.002	0.901, 1.113	0.976
		> 65 years	1.019	0.970, 1.072	0.452
	Model 3	≤ 65 years	1.001	0.897, 1.117	0.983
		> 65 years	1.029	0.975, 1.086	0.291
ATPase Inhibitory Factor 1^6^	Model 1	≤ 65 years	1.001	0.999, 1.003	0.278
		> 65 years	1.000	0.999, 1.002	0.572
	Model 2	≤ 65 years	1.001	0.999, 1.003	0.315
		> 65 years	1.000	0.998, 1.002	0.920
	Model 3	≤ 65 years	1.002	0.999, 1.004	0.154
		> 65 years	1.000	0.998, 1.002	0.942

≤ 65 years old *N* = 45; > 65 years *N* = 102. ^a^ *N* = 101. *No participant under 65 had cognitive impairment, so it was omitted from that model. ^1^Horvath Age acceleration, residuals after regressing chronological age on epigenetic age (adjusted for cell number), ^2^ Hannum Age acceleration, residuals after regressing chronological age on epigenetic age (adjusted for cell number), ^3^ PhenoAge acceleration, residuals after regressing chronological age on epigenetic age (adjusted for cell number), ^4^ GrimAge acceleration, residuals after regressing chronological age on epigenetic age, ^5^ Inflammatory Age acceleration, residuals after regressing chronological age on inflammatory age, ^6^ ATPase Inhibitory Factor 1 (IF1), measured in ng/mL in a subsample of 42 and 79 participants respectively

(Logistic Regression Models: Model 1 – Unadjusted; Model 2—Adjusted for BMI, Medications, and Weight Loss; Model 3 – Fully Adjusted for sex, Body Mass Index, Medications, Weight Loss, Depression Severity, and Cognitive Impairment*)

### Sex-stratified associations between biomarkers of aging and appetite loss

Table 4 presents the results for unadjusted and adjusted logistic regression, stratified by sex. After adjustments, a significant association was found between appetite loss and age acceleration for GrimAge in men (Odds Ratio = 2.09, 95% Confidence Interval: 1.26, 3.47). No significant associations between the biomarkers and appetite loss were found in women, although a nearly significant association was found for PhenoAge.

**Table 4:** Sex-stratified associations between epigenetic and inflammatory clocks, ATPase inhibitory factor 1, and appetite loss

	Unadjusted						Adjusted					
	Men *n* = 48			Women *n* = 99			Men *n* = 48			Women *n* = 99		
Biomarker of aging	Odds Ratio	95% CI for Odds Ratio	*P*-value	Odds Ratio	95% CI for Odds Ratio	*P*-value	Odds Ratio	95% CI for Odds Ratio	*P*-value	Odds Ratio	95% CI for Odds Ratio	*P*-value
Horvath Age Acceleration^1^	0.839	0.702, 1.003	0.054	1.049	0.964, 1.142	0.268	0.820	0.659, 1.021	0.076	1.084	0.969, 1.213	0.159
Hannum Age Acceleration^2^	0.854	0.710, 1.027	0.093	0.997	0.893, 1.113	0.958	0.945	0.763, 1.170	0.603	1.037	0.886, 1.213	0.653
PhenoAge Acceleration^3^	1.010	0.905, 1.128	0.854	1.111	1.014, 1.218	0.024	0.975	0.853, 1.115	0.714	1.132	0.997, 1.286	0.056
GrimAge Acceleration^4^	1.860	1.281, 2.699	0.001	1.090^a^	0.950, 1.251	0.219	2.094	1.263, 3.472	0.004	0.992^a^	0.824, 1.194	0.932
Inflammatory Age Acceleration^5^	1.000	0.937, 1.069	0.978	1.029	0.979, 1.082	0.255	1.038	0.946, 1.139	0.426	1.026	0.965, 1.090	0.417
ATPase Inhibitory Factor 1^6^	0.999	0.997, 1.002	0.761	1.001	0.999, 1.003	0.149	0.998	0.994, 1.002	0.324	1.002	0.999, 1.004	0.132

CI = Confidence Interval. ^a^ *n* = 98 people. ^1^ Horvath Age acceleration, residuals after regressing chronological age on epigenetic age (adjusted for cell number), ^2^ Hannum Age acceleration, residuals after regressing chronological age on epigenetic age (adjusted for cell number), ^3^ PhenoAge acceleration, residuals after regressing chronological age on epigenetic age (adjusted for cell number), ^4^ GrimAge acceleration, residuals after regressing chronological age on epigenetic age, ^5^ Inflammatory Age acceleration, residuals after regressing chronological age on inflammatory age, ^6^ ATPase Inhibitory Factor 1 (IF1), measured in ng/mL in a subsample of 40 men and 78 women

(Logistic Regression, adjusted for age, Body Mass Index, Medications, Weight Loss, Depression Severity and Cognitive Impairment)

## Discussion

The primary objective of this study was to examine the associations of biological aging markers with appetite loss in community-dwelling people aged 21 to 102 years. Consistent with our hypothesis, individuals with appetite loss exhibited accelerated biological aging, measured by the GrimAge clock, compared to people without appetite loss. Significant associations were not observed for any other age-related marker. In secondary analysis, significant associations between GrimAge acceleration and appetite loss were confirmed in individuals older than 65 years and men.

The observed association between appetite loss and age acceleration for the GrimAge clock may have several explanations. Research suggests that second-generation clocks are better at predicting clinical health decline related to aging compared to first-generation clocks (e.g., Horvath and Hannum clocks) [23]. One explanation for the observed association could be that the GrimAge clock is trained on proteins related to appetite regulation, such as leptin and growth differentiation factor 15 (GDF15) [9, 23]. Leptin is a well-known adipokine that plays a critical role in energy balance by signalling satiety to the hypothalamus and thereby reducing food intake [24]. Conversely, GDF15 is recognized as an anorexigenic protein that suppresses appetite and is often elevated in conditions associated with cachexia and aging [25]. Moreover, GDF15 levels have been linked to poor appetite and malnutrition, which could explain the observed association between appetite loss and age acceleration for GrimAge [26]. Given that GrimAge is based on these appetite-regulating proteins, it is plausible that it is particularly sensitive to age-related changes in appetite. Furthermore, appetite loss is a common symptom of aging and is often linked with a decline in physiological resilience, increased inflammation, and a higher disease burden, all of which are reflected by the GrimAge clock [9]. Consequently, GrimAge may better capture the complex interplay between appetite loss and accelerated aging compared to the other aging markers. Although no prior studies have directly examined the relationship between appetite loss and epigenetic clocks, our findings align with previous research linking GrimAge, more than other epigenetic aging clocks, to other age-related conditions, including frailty, cardiovascular disease, and mortality [9, 23, 27].

The GrimAge association with appetite loss was confirmed in people > 65 years and men, but not in individuals ≤ 65 and women. Regarding age, it is plausible that older individuals exhibit greater variability in age acceleration compared to younger adults, as observed in the entire INSPIRE-T cohort. This reflects the heterogeneity of aging and may enhance the sensitivity of associations between age acceleration and age-related clinical conditions. It is also possible that the absence of significant association in those ≤ 65 years is a statistical artefact due to an underpowered analysis, since the number of people was small. For sex, the sex-specific differences need further investigation, but since GrimAge is partly trained on leptin levels [9], which are strongly regulated by sex hormones [28], this may partly explain the findings. It is also possible that the factors influencing appetite loss vary by sex and that the clocks may capture the various aetiological factors of appetite loss differently in men and women. Indeed, cardiovascular diseases may lead to loss of appetite [29]; estrogen has protective cardiovascular effects [30], although this potential mechanism is less determinant in older postmenopausal women. Meanwhile, men accumulate more visceral fat, a strong determinant of cardiovascular conditions, than women during aging [31, 32].

Contrary to previous research linking inflammation to appetite loss [11, 15, 33], we found no significant association between appetite loss and inflammatory age acceleration, which is somewhat unexpected. Although this absence of association is difficult to explain, it is possible that the inflammatory age acceleration measured by the inflammatory age clock might not fully capture the specific inflammatory pathways directly involved in appetite regulation. Following that, the impact of inflammation on appetite is complex and may not always be straightforward. On one hand, pro-inflammatory cytokines such as IL-6 and TNF-α are known to reduce appetite by influencing hypothalamic pathways involved in appetite regulation [15, 33]. On the other hand, other inflammatory markers may have the opposite effect by reducing leptin sensitivity, potentially leading to decreased satiety, increased hunger and thereby an improved appetite [34]. This complexity is further highlighted by findings from Chareh et al. [15] who observed that while inflammation was linked to appetite loss, higher levels of leptin and inflammation were also associated with better appetite in some individuals. Even if leptin is traditionally known as a satiety hormone, its relationship with appetite is influenced by factors such as leptin resistance, which could alter the expected effects [34]. This suggests that the inflammation-appetite associations may vary and be dependent on individual features, such as metabolic health or underlying medical conditions. The links between appetite loss during aging and chronic inflammation need further investigation.

The absence of an association between IF1 and appetite loss was possibly influenced by the small sample size and the consequent lack of sufficient statistical power, since this analysis was conducted in a subsample of 121 participants due to data availability. In addition, IF1 in plasma does not exclusively reflect IF1 activity in the mitochondria, as IF1 is also found at the membrane of various cell types, where it may be involved in non-mitochondrial cellular processes and could be secreted [12, 35, 36]. Therefore, plasma IF1 may not provide accurate information about mitochondrial bioenergetics. Further studies looking at intracellular IF1 activity and appetite would shed light on the potential role mitochondrial bioenergetics may play in anorexia of aging.

### Strengths and limitations

A strength of this study is the case–control design, which matches participants by age and sex to reduce potential confounding effects. Unlike standard statistical adjustments, this approach ensures groups'comparability before analysis, rather than relying on corrections afterwards. This is particularly important for the present work since we investigate an age-related clinical condition using a dataset with a large age range (from 20 to 102 years at baseline); using the whole sample would have led to the constitution of a control group composed by younger adults (about 30% of the entire INSPIRE-T cohort is younger than 55 years-old), in whom appetite loss is uncommon. Additionally, to enhance the reliability of our findings, we applied conditional logistic regression to our case–control design, which accounts for the matched groups and helps control potential confounding factors.

The main limitation of this work is that appetite was assessed using the World Health Organization´s ICOPE screening tool, which relies on a single yes-or-no question. This approach may not fully capture the complexity of appetite loss as it does not take appetite severity into account and relies solely on subjective perceptions and definitions. Additionally, since both appetite assessment and some covariates (e.g., medications) were self-reported, this can lead to inaccurate information. Furthermore, the sample size for those reporting appetite loss was small (*n* = 49), which may limit statistical power. Future studies should consider including comorbidities that may influence appetite loss as covariates. Finally, adjustments for health-related covariates (e.g., cognitive function and depressive symptoms) were probably suboptimal since we opted not to use full-length scales to avoid losing cases due to data missing in those scales; future studies should consider using full-length scales for measuring appetite quality as well as key variables, such as cognitive function and depressive symptoms.

## Conclusion

This study identified a significant association between accelerated epigenetic aging for GrimAge and appetite loss. Future investigations using larger sample sizes and longitudinal study designs are needed to explore the relationship between biological age variation and changes in appetite. Additionally, studies using a more detailed, severity-based measure of appetite loss are needed to better understand these associations. This study, to our knowledge, is the first to investigate the link between biological aging markers and appetite loss, providing important insights and highlighting the need for further work in this domain.

## Supplementary Information

Below is the link to the electronic supplementary material.

Supplementary file1 (DOCX 27.6 KB)

Supplementary file2 (DOCX 30.1 KB)
