# Supplementary material for: The associations between biological markers of aging and appetite loss across adulthood: retrospective case–control data from the INSPIRE-T study
Source: GeroScience. 2025 May 10;48(1):859–70. doi: 10.1007/s11357-025-01691-w (PMC12972219; doi:10.1007/s11357-025-01691-w)
Supplement: Supplementary file 1 — Supplementary file1 (DOCX 27.6 KB) [file 11357_2025_1691_MOESM1_ESM.docx]

**Supplemental material**

| **Table A1** Details of the variables (sex and age) used for matching participants in a 1:2 ratio (two controls without appetite loss per one case with appetite loss) | | | |
| --- | --- | --- | --- |
| **Matching Group** | **Appetite^1^** | **Age** | **Sex^2^** |
|  | 0 | 30 | 1 |
| 1 | 0 | 30 | 1 |
|  | 1 | 30 | 1 |
|  | 0 | 59 | 1 |
| 2* | 0 | 58 | 1 |
|  | 1 | 59 | 1 |
|  | 0 | 61 | 1 |
| 3 | 0 | 61 | 1 |
|  | 1 | 61 | 1 |
|  | 0 | 61 | 1 |
| 4 | 0 | 61 | 1 |
|  | 1 | 61 | 1 |
|  | 1 | 65 | 1 |
| 5 | 0 | 65 | 1 |
|  | 0 | 65 | 1 |
|  | 0 | 71 | 1 |
| 6 | 0 | 71 | 1 |
|  | 1 | 71 | 1 |
|  | 0 | 72 | 1 |
| 7 | 0 | 72 | 1 |
|  | 1 | 72 | 1 |
|  | 0 | 79 | 1 |
| 8 | 0 | 79 | 1 |
|  | 1 | 79 | 1 |
|  | 1 | 80 | 1 |
| 9 | 0 | 80 | 1 |
|  | 0 | 80 | 1 |
|  | 0 | 80 | 1 |
| 10 | 0 | 80 | 1 |
|  | 1 | 80 | 1 |
|  | 1 | 81 | 1 |
| 11 | 0 | 81 | 1 |
|  | 0 | 81 | 1 |
|  | 0 | 85 | 1 |
| 12 | 0 | 85 | 1 |
|  | 1 | 85 | 1 |
|  | 0 | 86 | 1 |
| 13 | 1 | 86 | 1 |
|  | 0 | 86 | 1 |
|  | 0 | 87 | 1 |
| 14 | 0 | 87 | 1 |
|  | 1 | 87 | 1 |
|  | 0 | 21 | 2 |
| 15 | 1 | 21 | 2 |
|  | 0 | 21 | 2 |
|  | 0 | 30 | 2 |
| 16 | 0 | 30 | 2 |
|  | 1 | 30 | 2 |
|  | 0 | 31 | 2 |
| 17 | 1 | 31 | 2 |
|  | 0 | 31 | 2 |
|  | 1 | 55 | 2 |
| 18 | 0 | 55 | 2 |
|  | 0 | 55 | 2 |
|  | 1 | 56 | 2 |
| 19 | 0 | 56 | 2 |
|  | 0 | 56 | 2 |
|  | 0 | 61 | 2 |
| 20 | 0 | 61 | 2 |
|  | 1 | 61 | 2 |
|  | 0 | 61 | 2 |
| 21 | 1 | 61 | 2 |
|  | 0 | 61 | 2 |
|  | 0 | 63 | 2 |
| 22 | 1 | 63 | 2 |
|  | 0 | 63 | 2 |
|  | 1 | 65 | 2 |
| 23 | 0 | 65 | 2 |
|  | 0 | 65 | 2 |
|  | 0 | 65 | 2 |
| 24 | 1 | 65 | 2 |
|  | 0 | 65 | 2 |
|  | 0 | 71 | 2 |
| 25 | 0 | 71 | 2 |
|  | 1 | 71 | 2 |
|  | 0 | 74 | 2 |
| 26 | 0 | 74 | 2 |
|  | 1 | 74 | 2 |
|  | 0 | 75 | 2 |
| 27 | 0 | 75 | 2 |
|  | 1 | 75 | 2 |
|  | 1 | 75 | 2 |
| 28 | 0 | 75 | 2 |
|  | 0 | 75 | 2 |
|  | 1 | 76 | 2 |
| 29 | 0 | 76 | 2 |
|  | 0 | 76 | 2 |
|  | 0 | 77 | 2 |
| 30 | 1 | 77 | 2 |
|  | 0 | 77 | 2 |
|  | 0 | 79 | 2 |
| 31 | 0 | 79 | 2 |
|  | 1 | 79 | 2 |
|  | 0 | 79 | 2 |
| 32 | 0 | 79 | 2 |
|  | 1 | 79 | 2 |
|  | 0 | 80 | 2 |
| 33 | 0 | 80 | 2 |
|  | 1 | 80 | 2 |
|  | 1 | 83 | 2 |
| 34 | 0 | 83 | 2 |
|  | 0 | 83 | 2 |
|  | 1 | 83 | 2 |
| 35 | 0 | 83 | 2 |
|  | 0 | 83 | 2 |
|  | 0 | 83 | 2 |
| 36 | 0 | 83 | 2 |
|  | 1 | 83 | 2 |
|  | 0 | 84 | 2 |
| 37 | 0 | 84 | 2 |
|  | 1 | 84 | 2 |
|  | 0 | 84 | 2 |
| 38 | 0 | 84 | 2 |
|  | 1 | 84 | 2 |
|  | 0 | 85 | 2 |
| 39* | 0 | 84 | 2 |
|  | 1 | 84 | 2 |
|  | 0 | 84 | 2 |
| 40 | 0 | 84 | 2 |
|  | 1 | 84 | 2 |
|  | 0 | 86 | 2 |
| 41 | 1 | 86 | 2 |
|  | 0 | 86 | 2 |
|  | 0 | 86 | 2 |
| 42 | 0 | 86 | 2 |
|  | 1 | 86 | 2 |
|  | 0 | 86 | 2 |
| 43 | 0 | 86 | 2 |
|  | 1 | 86 | 2 |
|  | 1 | 87 | 2 |
| 44 | 0 | 87 | 2 |
|  | 0 | 87 | 2 |
|  | 1 | 88 | 2 |
| 45 | 0 | 88 | 2 |
|  | 0 | 88 | 2 |
|  | 0 | 92 | 2 |
| 46 | 0 | 92 | 2 |
|  | 1 | 92 | 2 |
|  | 0 | 93 | 2 |
| 47 | 1 | 93 | 2 |
|  | 0 | 93 | 2 |
|  | 1 | 91 | 1 |
| 48* | 0 | 90 | 1 |
|  | 0 | 90 | 1 |
|  | 0 | 90 | 1 |
| 49** | 1 | 102 | 1 |
|  | 0 | 90 | 1 |
| 1: 0: no appetite loss, 1: appetite loss.  2: 1: Male, 2: Female  * Matched with a maximum one-year age difference  ** 102-year-old male was matched with two controls aged 90, as no closer matches by sex and age were available | | | |
