# Supplementary material for: The associations between biological markers of aging and appetite loss across adulthood: retrospective case–control data from the INSPIRE-T study
Source: GeroScience. 2025 May 10;48(1):859–70. doi: 10.1007/s11357-025-01691-w (PMC12972219; doi:10.1007/s11357-025-01691-w)
Supplement: Supplementary file 2 — Supplementary file2 (DOCX 30.1 KB) [file 11357_2025_1691_MOESM2_ESM.docx]

**Supplementary Material - Sensitivity Analysis: Exclusion of 102-Year-Old Case and Matched Controls (N = 144)**

| **Table A2. Differences in Characteristics, Epigenetic and Inflammatory Clocks and ATPase Inhibitory Factor 1 Between Cases and Controls (n=144)** | | | |
| --- | --- | --- | --- |
| **Variable** | **Cases (n=48)** | **Controls (n=96)** | ***P*-value** |
| **Age *** | 79 (20) | 79 (20) | 0.997 |
| **Sex *** |  |  | 1.000 |
| Women | 33 (68.75%) | 66 (68.75%) |  |
| **Body Mass Index** | 24.8 (6.36) | 24.4 (5.28) | 0.941 |
| **Medications/Day** | 6 (7) | 2 (5) | <0.001 |
| **Recent weight loss** |  |  | 0.009 |
| >3 kg loss (last 3 months) | 10 (20.83%) | 6 (6.25%) |  |
| No weight loss | 38 (79.17%) | 90 (93.75%) |  |
| **Depressive signs** |  |  | <0.001 |
| Yes | 32 (66.67%) | 24 (25.00%) |  |
| No | 16 (33.33%) | 72 (75.00%) |  |
| **Cognitive impairment** |  |  | 0.784 |
| Yes | 2 (4.17%) | 5 (5.21%) |  |
| No | 46 (95.83%) | 91 (94.79%) |  |
| **Biomarker of aging** |  |  |  |
| Horvath Age Acceleration^1^ | -0.80 (6.62) | -0.44 (5.97) | 0.936 |
| Hannum Age Acceleration^2^ | -1.25 (5.69) | -0.77 (4.57) | 0.492 |
| PhenoAge acceleration^3^ | 0.98 (6.23) | -0.77 (7.10) | 0.045 |
| GrimAge Age Acceleration^4^ | 1.20 (7.35) | -1.31 (3.75) ^a^ | 0.018 |
| Inflammatory Age acceleration^5^ | 2.01 (14.21) | 1.18 (14.18) | 0.482 |
| ATPase Inhibitory Factor 1^6^ (ng/mL) | 490.6 (322.9) ^c^ | 508.4 (252.8) ^b^ | 0.867 |
| ^a^ n=95, ^b^ n=81, ^c^ n=39.  Chi-Square Test and Fisher´s Exact Test (cell frequency<5) were used for categorical variables (n (%)). Wilcoxon Rank-Sum Test (median (interquartile range) was used for continuous variables (age, BMI, medications, and biomarkers). * Cases and controls matched on age and sex. ^1^ Horvath Age acceleration, residuals after regressing chronological age on epigenetic age (adjusted for cell number), ^2^ Hannum Age acceleration, residuals after regressing chronological age on epigenetic age (adjusted for cell number), ^3^ PhenoAge acceleration, residuals after regressing chronological age on epigenetic age (adjusted for cell number), ^4^ GrimAge acceleration, residuals after regressing chronological age on epigenetic age, ^5^ Inflammatory Age acceleration, residuals after regressing chronological age on inflammatory age, ^6^ ATPase Inhibitory Factor 1 (IF1), measured in ng/mL in a subsample of 120 participants. | | | |

| **Table A3. Associations Between Epigenetic and Inflammatory Clocks, ATPase Inhibitory Factor 1, and Appetite Loss** (Conditional Logistic Regression, Adjusted for Body Mass Index, Medications, Weight Loss, Depression Severity and Cognitive Impairment) | | | | | | |
| --- | --- | --- | --- | --- | --- | --- |
|  | **Unadjusted Model  N cases=48, N controls=96** | | | **Adjusted Model**  **N cases=48, N controls=96** | | |
| **Biomarker of aging** | **Odds Ratio** | **95% CI for  Odds Ratio** | ***P*-value** | **Odds Ratio** | **95% CI for Odds Ratio** | ***P*-value** |
| Horvath Age Acceleration^1^ | 0.992 | 0.924, 1.066 | 0.835 | 1.003 | 0.913, 1.102 | 0.942 |
| Hannum Age Acceleration^2^ | 0.955 | 0.872, 1.046 | 0.326 | 0.983 | 0.864, 1.118 | 0.794 |
| PhenoAge Acceleration^3^ | 1.074 | 1.000, 1.154 | 0.050 | 1.063 | 0.970, 1.164 | 0.191 |
| GrimAge Acceleration^4^ | 1.190^a^ | 1.055, 1.343 | 0.005 | 1.220^a^ | 1.032, 1.439 | 0.020 |
| Inflammatory Age Acceleration^5^ | 1.017 | 0.976, 1.060 | 0.416 | 1.044 | 0.985, 1.107 | 0.144 |
| ATPase Inhibitory Factor 1^6^ | 1.000 | 0.999, 1.002 | 0.280 | 1.001 | 0.999, 1.003 | 0.244 |
| CI= Confidence Interval. ^a^ n=48 cases, n=95 controls. ^1^ Horvath Age acceleration, residuals after regressing chronological age on epigenetic age (adjusted for cell number), ^2^ Hannum Age acceleration, residuals after regressing chronological age on epigenetic age (adjusted for cell number), ^3^ PhenoAge acceleration, residuals after regressing chronological age on epigenetic age (adjusted for cell number), ^4^ GrimAge acceleration, residuals after regressing chronological age on epigenetic age, ^5^ Inflammatory Age acceleration, residuals after regressing chronological age on inflammatory age, ^6^ ATPase Inhibitory Factor 1 (IF1), measured in ng/mL in a subsample of 105 participants. | | | | | | |

| **Table A4. Age-Stratified Associations Between Epigenetic and Inflammatory Clocks, ATPase Inhibitory Factor 1, and Appetite Loss** (Logistic Regression, adjusted for sex, Body Mass Index, Medications, Weight Loss, Depression Severity and Cognitive Impairment*) | | | | | | | | | | | | |
| --- | --- | --- | --- | --- | --- | --- | --- | --- | --- | --- | --- | --- |
|  | **Unadjusted** | | | | | | **Adjusted** | | | | | |
|  | **Age ≤ 65 years old N=45** | | | **Age > 65 years N=99** | | | **Age ≤ 65 years old N=45** | | | **Age > 65 years N=99** | | |
| **Biomarker of aging** | **Odds ratio** | **95% CI for Odds Ratio** | ***P*-value** | **Odds ratio** | **95% CI for Odds Ratio** | ***P*-value** | **Odds ratio** | **95% CI for Odds Ratio** | ***P*-value** | **Odds Ratio** | **95% CI for Odds Ratio** | ***P*-value** |
| Horvath Age  Acceleration^1^ | 1.066 | 0.918, 1.238 | 0.400 | 0.973 | 0.898, 1.053 | 0.495 | 1.029 | 0.847, 1.250 | 0.775 | 0.988 | 0.892, 1.095 | 0.817 |
| Hannum Age  Acceleration^2^ | 1.158 | 0.913, 1.468 | 0.227 | 0.920 | 0.829, 1.021 | 0.116 | 1.168 | 0.873, 1.563 | 0.295 | 0.956 | 0.830, 1.100 | 0.527 |
| PhenoAge  Acceleration^3^ | 1.110 | 0.936, 1.317 | 0.231 | 1.061 | 0.984, 1.145 | 0.121 | 1.090 | 0.883, 1.347 | 0.421 | 1.066 | 0.971, 1.170 | 0.179 |
| GrimAge  Acceleration^4^ | 1.041 | 0.854, 1.270 | 0.691 | 1.214^a^ | 1.065, 1.385 | 0.004 | 0.995 | 0.763, 1.299 | 0.973 | 1.352^a^ | 1.103, 1.655 | 0.004 |
| Inflammatory Age Acceleration^5^ | 0.991 | 0.906, 1.085 | 0.852 | 1.023 | 0.978, 1.070 | 0.326 | 1.001 | 0.897, 1.117 | 0.983 | 1.024 | 0.970, 1.081 | 0.392 |
| ATPase Inhibitory  Factor 1^6^ | 1.001 | 0.999, 1.003 | 0.278 | 1.000 | 0.999, 1.002 | 0.642 | 1.002 | 0.999, 1.004 | 0.154 | 1.000 | 0.998, 1.002 | 0.995 |
| *No participant under 65 had cognitive impairment, so it was omitted from that model. CI= Confidence Interval. ^a^ n=98 people. ^1^Horvath Age acceleration, residuals after regressing chronological age on epigenetic age (adjusted for cell number), ^2^ Hannum Age acceleration, residuals after regressing chronological age on epigenetic age (adjusted for cell number), ^3^ PhenoAge acceleration, residuals after regressing chronological age on epigenetic age (adjusted for cell number), ^4^ GrimAge acceleration, residuals after regressing chronological age on epigenetic age, ^5^ Inflammatory Age acceleration, residuals after regressing chronological age on inflammatory age, ^6^ ATPase Inhibitory Factor 1 (IF1), measured in ng/mL in a subsample of 42 and 78. | | | | | | | | | | | | |

| **Table A5. Sex-Stratified Associations between Epigenetic and Inflammatory Clocks, ATPase Inhibitory Factor 1, and Appetite Loss** (Logistic Regression, adjusted for age, Body Mass Index, Medications, Weight Loss, Depression Severity and Cognitive Impairment) | | | | | | | | | | | | |
| --- | --- | --- | --- | --- | --- | --- | --- | --- | --- | --- | --- | --- |
|  | **Unadjusted** | | | | | | **Adjusted** | | | | | |
|  | **Men n=45** | | | **Women n=99** | | | **Men n=45** | | | **Women n=99** | | |
| **Biomarker of aging** | **Odds Ratio** | **95% CI for Odds Ratio** | ***P*-value** | **Odds Ratio** | **95% CI for Odds Ratio** | ***P*-value** | **Odds Ratio** | **95% CI for Odds Ratio** | ***P*-value** | **Odds Ratio** | **95% CI for Odds Ratio** | ***P*-value** |
| Horvath Age Acceleration^1^ | 0.829 | 0.690, 0.997 | 0.046 | 1.049 | 0.964, 1.142 | 0.268 | 0.817 | 0.654, 1.020 | 0.074 | 1.084 | 0.969, 1.213 | 0.159 |
| Hannum Age Acceleration^2^ | 0.846 | 0.698, 1.026 | 0.089 | 0.997 | 0.893, 1.113 | 0.958 | 0.926 | 0.745, 1.152 | 0.491 | 1.037 | 0.886, 1.213 | 0.653 |
| PhenoAge  Acceleration^3^ | 1.010 | 0.903, 1.131 | 0.857 | 1.111 | 1.014, 1.218 | 0.024 | 0.964 | 0.836, 1.111 | 0.613 | 1.132 | 0.997, 1.286 | 0.056 |
| GrimAge  Acceleration^4^ | 1.924 | 1.291, 2.868 | 0.001 | 1.090^a^ | 0.950, 1.251 | 0.219 | 2.336 | 1.293, 4.221 | 0.005 | 0.992^a^ | 0.824, 1.194 | 0.932 |
| Inflammatory Age Acceleration^5^ | 0.992 | 0.927, 1.062 | 0.817 | 1.029 | 0.979, 1.082 | 0.255 | 1.022 | 0.931, 1.123 | 0.642 | 1.026 | 0.965, 1.090 | 0.417 |
| ATPase Inhibitory  Factor 1^6^ | 0.999 | 0.997, 1.002 | 0.634 | 1.001 | 0.999, 1.003 | 0.149 | 0.998 | 0.994, 1.002 | 0.281 | 1.002 | 0.999, 1.004 | 0.132 |
| CI= Confidence Interval. ^a^ n=98 people. ^1^ Horvath Age acceleration, residuals after regressing chronological age on epigenetic age (adjusted for cell number), ^2^ Hannum Age acceleration, residuals after regressing chronological age on epigenetic age (adjusted for cell number), ^3^ PhenoAge acceleration, residuals after regressing chronological age on epigenetic age (adjusted for cell number), ^4^ GrimAge acceleration, residuals after regressing chronological age on epigenetic age, ^5^ Inflammatory Age acceleration, residuals after regressing chronological age on inflammatory age, ^6^ ATPase Inhibitory Factor 1 (IF1), measured in ng/mL in a subsample of 40 men and 80 women. | | | | | | | | | | | | |
